# *Acacia koa* seedling disease tolerance and vigor driven by breeding orchard size

**DOI:** 10.3389/fpls.2025.1544491

**Published:** 2025-05-08

**Authors:** Nathan Fumia, Nicklos Dudley, Tyler Jones, John Dobbs, Jane Stewart, Michael Kantar

**Affiliations:** ^1^ Department of Tropical Plant and Soil Sciences, University of Hawaii at Manoa, Honolulu, HI, United States; ^2^ Crop Improvement Division, Hawaii Agriculture Research Center, Waipahu, HI, United States; ^3^ Department of Agricultural Biology, Colorado State University, Fort Collins, CO, United States

**Keywords:** tree breeding, sequential thinning, recurrent selection, *Fusarium oxysporum*, stochastic simulation

## Abstract

*Acacia koa* Gray (koa) is a Hawaiian endemic tree species that has a long history of use in the islands. In the late 20th century disease started impacting native koa stands, leading to the initiation of seed orchards that were founded from seeds collected across the islands. Large improvements in disease tolerance and vigor were achieved in very few cycles of selection despite the long temporal time of this perennial hardwood tree species. Initial selection on agronomic and domestication traits improved populations to the agricultural and natural ecosystem. Further, using simulation we identified how different methods of selection could be implemented to more rapidly make progress toward improved koa germplasm. Our evidence shows that domestication in *Acacia koa* provides a model for parametrization of crossing in the breeding cycle for rapid improvement of any tree species.

## Introduction

*Acacia koa* Gray (hereafter, koa) is an endemic timber tree species of the Hawaiian archipelago with important cultural and ecological relevance (Baker, 2009). Historically used for ocean-voyaging canoes, koa is presently used as a high value input in fine furniture, jewelry, and musical instruments. Past deforestation for agriculture and ranching during the 19th and 20th centuries, along with its exquisite curly grain and rich coloration has driven the price to $125 per board foot and estimated annual gross value of $20–30 million (Yanagida et al., 2004). Further, koa is a critical canopy tree providing habitat for endangered native birds and epiphytic plants (Pejchar et al., 2005; Baker, 2009).

Koa is an obligate outcrossing (cross-pollinating), autotetraploid species (2n=52) (Carr, 1978; Shi, 2003). The species arose from a genome duplication event of the Australian species *Acacia melanoxylon* Brown (Brown et al., 2012; Le Roux et al., 2014). Colonization of the Hawaiian archipelago by koa likely occurred millions of years ago based on pollen records (Hotchkiss and Juvik, 1999) and host-specific endemic insect species (Gagne, 1979). Phenotypic and genotypic variation is found between and within different populations through the Hawaiian Islands (Wagner et al., 1999; Sun, 1996; Adamski et al., 2012; Dudley et al., 2020). Variation can also be found by eco-region (primarily wet windward or dry leeward populations) and elevation, yet these sub-groups still hierarchically cluster by island (Dudley et al., 2017).

Compounding the impact of deforestation on population decline is the rise of *Fusarium oxysporum f.* sp. *Koae* (FOXY), commonly known as koa wilt disease (Gardner, 1980; Anderson et al., 2002; Dudley et al., 2007), decimating the remaining forest by clogging xylem flow of infected, susceptible trees. However, varying levels of resistance to the disease-causing pathogen can be found in each population of koa (Dudley et al., 2015) prompting investigation into disease resistant seed production for koa orchard and reforestation efforts (Dudley et al., 2020). The cultural, ecological, and economic importance of koa is the driving force behind facilitating range expansion of the species by increasing tolerance to the disease in distinct koa populations, providing a mechanism by which koa realizes a domesticated state capable of cultivation in the agroecosystems and restoration of the ecosystems of Hawaii. The traits essential for domestication vary depending on the organism, its life-history and likely agroecosystem. Koa, as a long-lived perennial with agricultural and ecological significance, requires traits that facilitate range expansion, one of which is the ability to tolerate or resist infection by FOXY. Although disease resistance typically has simple genetic architecture (Jungers et al., 2023), tolerance is often proxied through improvements in different more complex traits, such as vigor. Previous work in Koa has been unable to find major effect disease resistance for FOXY (Dudley et al., 2015). However, during the ongoing breeding efforts there have been many reports of tolerance (Dudley et al., 2007, 2015, 2017). Typically a tolerance response is associated with more complex inheritance, this also follows work on FOXY in several plant species, such as Lycopersicon (Li et al., 2022), Arabidopsis (Diener and Ausubel, 2005) and Fragaria (Pincot et al., 2022).

Population improvement through artificial selection requires optimization of genetic gain for different traits and is tailored towards the end goal (Gaynor et al., 2017). This is achieved through an iterative process of generational increase in favorable alleles in the population under selection, acting to increase the probability of extracting a superior cultivar from the population (Cobb et al., 2019; Van Tassel et al., 2020). The increase in favorable alleles drives genetic gain, which is a product of additive genetic variation within the population, selection intensity, and selection accuracy. Each gain component can be increased through different methods and technology applied across the breeding cycle (Hickey et al., 2017; Wallace et al., 2018; Wartha and Lorenz, 2021). Population improvement relies upon adequate parametrization in the breeding cycle of crossing, evaluation, and selection (CES; Covarrubias-Pazaran et al., 2022).

The focus of this study is the effect of crossing on a wild species being selected for a domesticated form, including decisions such as number of parents, number of crosses, and number of progeny to continue. Moreover, this accounts for the interplay of traits, seedling vigor and disease resistance – a common occurrence during neo-domestication. Here general knowledge on how to begin breeding scheme development for wild species (neo-domestication) is developed through the use of koa as a novel system to understand crossing and breeding population size during incipient domestication. The alteration of breeding population size in the koa orchard is through thinning of individuals, a form of pollen control in the population. These individuals are thinned for varying reasons, one of which being a low durability of resistance which causes trees to succumb to disease over time. As the breeding population size decreases, and therefore diminished background genetic variability, the survival probability is increased by removal of susceptible types from the breeding population. This is an opportunity for insight to develop expectations of population improvement through augmented crossing parameters to inform situational changes through breeding cycles in neo-domestication programs (Cobb et al., 2019; Covarrubias-Pazaran et al., 2022).

## Materials and methods

### Germplasm and breeding

Individual wild accessions were sourced from all major islands in the Hawaiian archipelago (Big Island, Maui, Oahu, Kauai) at dry-leeward and wet-windward locations in the late 1990s and early 2000s where seed was grouped into 120 half-sibling bulk seed families from Oahu (**Supplementary Figure 1**). Disease screening following methods reported in Dudley et al. (2015) were completed for each half-sibling bulk where maternal lineages were removed for low progeny disease resistance out of 24 total seedlings per family (proportion survival <60%). Resistant maternal half-sibling seedlings (proportion survival >60%) were then used in the randomized complete block design orchard in 2012. This orchard includes 24 half-sibling seeds from 35 maternal families, including one known susceptible family, using 4 blocks and 6 trees per plot, for a total of 840 individuals. It was not until 5 years later that seed was collected, following the first orchard thinning (2017). Thinning in the orchard is done to remove individuals with poor tree structure, low diameter at breast height (DBH), low durability of resistance, and low or no seed production. This process has been repeated three more times for a second (2018), third (2019), and fourth thinning (2021) with subsequent seed collections (**Supplementary Figure 1**).

### Trials

To quantify the effect of breeding population size on seedling disease resistance, bulk seed from each thinning group (1^st^, 2^nd^, 3^rd^, 4^th^) were placed into a randomized complete block design in the greenhouse for disease screening compared against control seed (wild collection from native range – Ko’olau Mountains, Oahu). Variable numbers of seed were used for germination processes from each group: 480 seeds from wild sources (collected 2012), 347 seeds in thinning 1 (collected 2017), 342 seeds in thinning 2 (collected 2018), 306 seeds in thinning 3 (collected 2019), and 206 seeds in thinning 4 (collected 2021). Seed is scarified through snipping, soaked for 24-hours in distilled water, and planted in seedling trays until 1-inch in height. Furthermore, variable germination rates were observed by seed group, owing to the variable ages of the seed: 13.5% in wild source (59 plants), 42.9% in thinning 1 (139 plants), 80.7% in thinning 2 (200 plants), 89.2% in thinning 3 (200 plants), and 93.8% in thinning 4 (200 plants). Once koa seed reaches 1-inch in height, the germinated seed is placed into inoculated (treatment) or sterile (control) media and watered. Disease screening methods follow those used in the late 1990s and described during wild pathogenicity trials reported in Dudley et al. (2007). Prior to mixing with growing media, isolates were grown on potato dextrose agar in a single petri dish per race. Each FOXY race was then separately grown for inoculum using a combination of perlite, cornmeal, and potato dextrose agar. Once fully colonized, the inoculum was ground to a fine powder and equal parts of all 9 races were homogenized and mixed thoroughly with the growing media for the FOXY treated split-plots. The inoculum was a cocktail of 9 highly virulent races of FOXY, identified and isolated by Dr. Susan Schencke and Nick Dudley to estimate broad tolerance to the variety of virulent races likely to be encountered in the agroecosystem (Dudley et al., 2007). The individual plants are then held growing in their respective location until visual signs, scored on a weekly basis, of wilt and/or death is observed.

Death by FOXY is quantified by including a split-plot to our RCBD where half of the seedlings per plot were planted into inoculated media and half into sterile media. Therefore, proportion of group survival will be used as the measure of disease resistance per population. Our experimental design follows by having 4 blocks, 5 plots, and 2 splits per plot with 25 plants per split in thinnings 2, 3, and 4, 17–18 plants per split in thinning 1, and 7–8 plants per split in wild source.

Koa seedlings from each seed group and split were also sampled for pathogen confirmation. Each of the 33 sampled seedlings had its root collar, which included a small portion of the root and stem, severed and surface sterilized with 10% Clorox bleach solution and rinsed twice in sterile water. These segments were transferred to ¼ potato dextrose agar (PDA) petri plates and incubated at 25°C for 3 days. Once mycelial growth was observed from each sample, hyphal tips were taken for each seedling that appeared to be *Fusarium*. These cultures were grown for a week to get sufficient growth of mycelium for storage. Representative cultures were grown in potato dextrose broth (PDB) for 7 days for extraction of total DNA using a Zymo plant/fungal DNA extraction kit. Polymerase chain reaction (PCR) was used to amplify the rpb2 locus for these isolates and sent for sanger sequencing in both the forward and reverse directions at Eurofins genomics. Sequences were trimmed and aligned using Geneious prime and consensus sequences were BLASTed to the NCBI GenBank database for identification. The primers used are for the locus RNA polymerase II subunit (Liu et al., 1999) and have been used in previous studies to differentiate *Fusarium* spp. (**Supplementary Table 1**; Dobbs et al., 2020).

### Analysis

Data collected during this trial include a weekly survey of death (timing of death) and seedling height at trial end (120 days). To investigate the effect of breeding population size on progeny seedling disease resistance, we employ binomial regression with a logit-link function through a generalized mixed-effects model (Bates et al., 2015) to make predictions about the probability of survival of a given seed dependent on its source group. The formula uses the ratio of plants at the start of the trial to total death as the response variable with random predictor of block and fixed predictors seed group and split treatment. Next, we use a two-sample t-test to compare means of control and fusarium treated (split treatment) because disease pressure degrades plant vigor, especially affecting the seedling stage. Moving past the t-test, we apply a linear mixed-model approach to estimate seed group and treatment effects on seedling vigor at 120 days (height in mm) while removing random blocking effects. Statistical analysis and visualization have been performed in the R environment (R Core Team, 2023).

### Simulation integration and comparison

Once mixed-model analysis estimates seed group effect and predicts residual errors, we move to integrate to stochastic simulation (AlphaSimR; Gaynor et al., 2021) for future projection and estimating genetic complexities. More specifically, we adjust the founding population parameters to match estimates of our different seed sources to detangle the influence of population size and resistance on seedling vigor. For future projection, we simulate alternative genetic complexities for seedling vigor as simple oligogenic (8 QTL), complex oligogenic (20 QTL), and polygenic (100 QTL) over 10 cycles of selection. Designing the founders takes species specific information such as 13 chromosomes in koa, a genome duplication to simulate autotetraploidy, as well as specifying mean and variance for the trait along each seed group (Wild through GroupE, **Table 1**). Once a trait is specified under a given genetic architecture, phenotypes are estimated by taking into account the additive genetic architecture with the residual error, estimated through mixed-model (**Table 1**). We then specify factors relevant to the breeding cycle, such as number of parents (# of maternal parents for each group, **Table 1**), number of crosses per parent (parents-1), and number of progeny from each parent (varied according to number of parents to have ~500 total progeny). Number of parents follows empirical seed group amounts (**Table 1**) while number of progeny per parent is altered to maintain ~500 total progeny each cycle (Wild: 53x10; First: 43x12; Second: 18x28, Third: 17x30; Fourth: 16x32). The founding population (cycle 0) therefore matches wild seed group linear mixed-model estimates while each seed groups linear mixed-model estimates form cycle 1 and serve as the backbone for 10 cycles of selection within each group. The 10 cycles of selection are selected using truncation selection for the number of parents with the highest phenotypic observation. But, important to this simulation is the presence of disease, which we simulate through random selection and removal of breeding candidates for death at the proportion of death (1-survival probability). The simulation is replicated 100 times and the mean (per cycle) phenotypic value and variance, along with their respective standard errors, are output.

**Table 1:** Linear mixed-model estimates of vigor (seedling height at 120 days post-germination) under control (untreated) and fusarium (treated) conditions.

LMM Coefficients	Seed Group (Maternal #)	Vigor Control Estimate (SE)	Vigor Fusarium Estimate (SE)	Vigor Fusarium Estimate
*Intercept*	Wild Seed 2012 (53)	23.112 (0.850)	-3.833 (0.392)	19.279
*B Effect*	1st Thinning (43)	-1.371 (0.923)		17.908
*C Effect*	2nd Thinning (18)	-2.516 (0.882)		16.763
*D Effect*	3rd Thinning (17)	-2.215 (0.882)		17.064
*E Effect*	4th Thinning (16)	-0.592 (0.880)		18.687

We move to investigate the effects of breeding population size, number of progeny, and survival probability on seedling vigor gain through 10 cycles of selection. To do so, we select polygenic (100 QTL) to serve as the genetic architecture of seedling vigor, with founding population and cycle 1 being formed the same way as previously but altering number of parents, progeny, and survival probability. Therefore, we set 3 comparative schemes: (1) seed group survival probabilities remain the same as empirical while setting constant - number of parents (43) and progeny (12); (2) seed group survival probabilities remain the same as empirical while setting constant – number of parents (16) and progeny (32); and (3) seed group survival probabilities are inverted (First to Fourth, Second to Third, vice versa) and seed group number of parents and progeny remain the same as empirical. These alterations will inform our understanding of the effect of survival probability on vigor gain through 10 cycles of selection and the effect of breeding population size to mitigate these effects.

## Results

**Figure 1:**
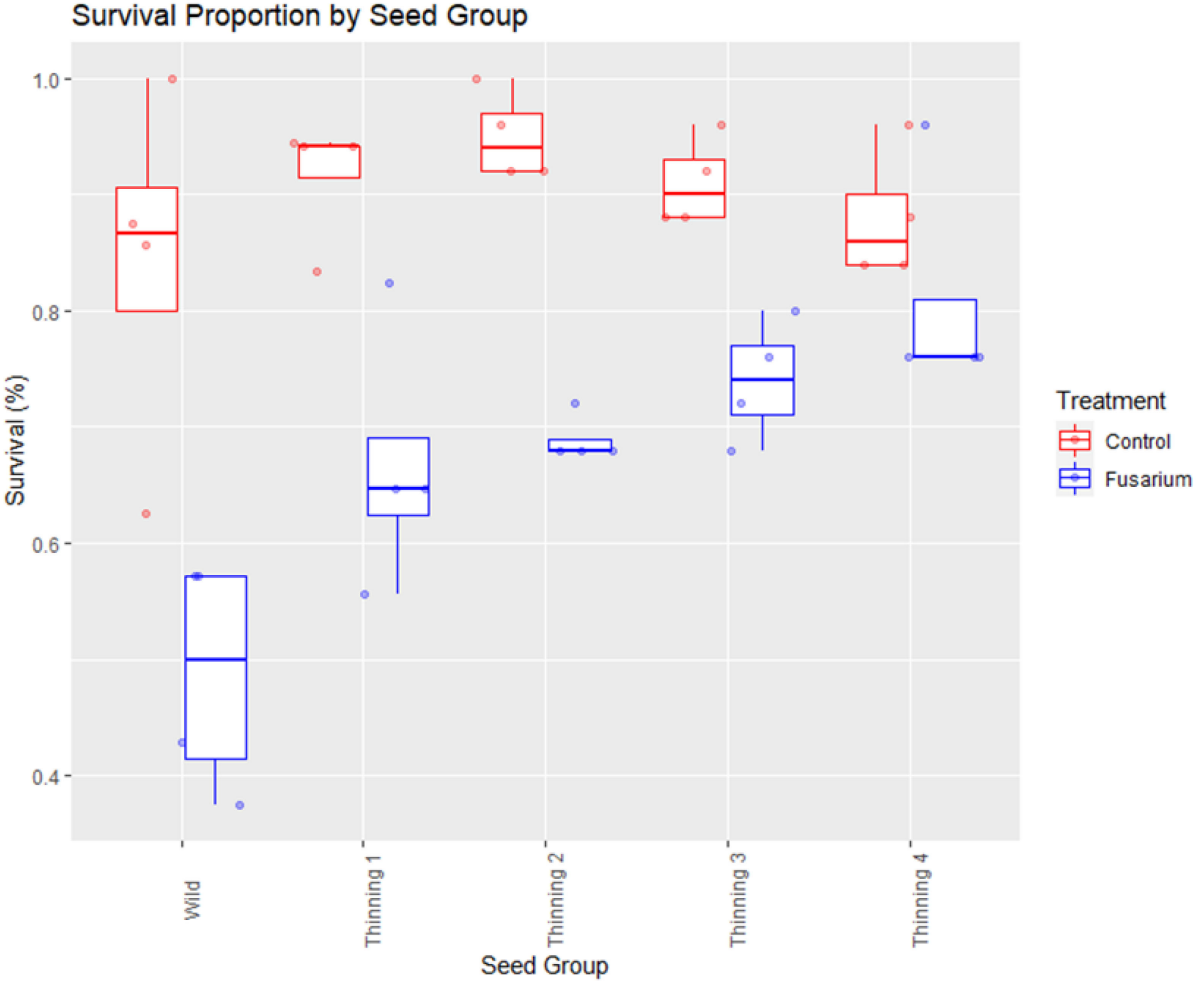
Proportion of seedling survival by seed source group. Comparison of control split (no FOXY applied) and fusarium (FOXY applied) tolerance.

The size and status of a breeding population has a direct influence on the frequency of important traits like disease tolerance, vigor, and survivability in progeny seedlings. Differences between the control and disease treatments were observed during trial execution in survival and vigor. The proportion of survival in control groups ranged from 85-95%, but with the application of FOXY to media during planting there is variable effect by thinning group (**Figure 1**). Wild source seed has the lowest tolerance to disease (mean: 50%) while the seed collected following the 4^th^ thinning of the seed orchard has the highest (mean: 75%). Each seed group between the wild and the 4^th^ thinning exhibit incrementally improved survival (**Figure 1**).

Koa seedlings sent for pathogen confirmation included 33 samples total with at least 2 samples per seed group and split (**Supplementary Table 2**). Three of the groups (Groups A, D, & E) results were as expected, with 0% of the control samples and 100% of the treated samples found FOXY. The other two groups had variable results. Group C found 0% of the control samples but only 80% of the treated samples found FOXY. Furthermore, Group B found 33% (1 sample) of the control samples and 67% of the treated samples with FOXY.

### Case Study of koa domestication

#### Generalized mixed-model estimates of survival probability

Generalized binomial mixed-effects model identifies seed from the second, third, and fourth thinning as being significantly higher proportion survival as compared to the intercept (wild seed; p<0.05). However, estimates of these groups are not different from one another, improving log-odds by 0.66 (0.32), 0.67 (0.32), and 0.77 (0.33) over the wild seed intercept of 1.79 (0.30), respectively (**Table 2**). Furthermore, when we apply the logistic link function to the log-odds, control probability of survival is 85.7% in wild seed with the largest increase in survival of the 5-year-old orchard seedlings (1^st^ thinning, Group B) to 90.8% (**Table 2**). Probability of survival is further increased in the 6-year-old orchard seedlings (2^nd^ thinning, Group C) to 92.0% with minimal increase in following years and orchard thinnings to the 9-year-old orchard seedlings (4^th^ thinning, Group E) to 92.8%. Application of FOXY (treatment) reduces predicted log-odds of survival by 1.13 (0.20; p<0.0001). Logistic link function finds treatment effect of fusarium disease probability of survival dropping in every group, with the largest decrease in survival estimated in wild seed by 20% to 65.9% survival and the smallest decrease in survival estimated in the 9-year-old orchard seedlings (4^th^ thinning, Group E) by 12% to 80.7% survival (**Table 2**).

**Table 2:** Generalized linear mixed-model log-odds estimates and probability of survival by seed group under control (untreated) and fusarium (treated) conditions.

GLMM Coefficients	Seed Group (Maternal #)	LogOdds Estimate (SE)	Probability Control	LogOdds Fusarium Estimate (SE)	Probability Fusarium
*Intercept*	Wild Seed 2012 (53)	1.790 (0.301)	0.857	-1.130 (0.203)	0.659
*B Effect*	1st Thinning (43)	0.498 (0.338)	0.908		0.761
*C Effect*	2nd Thinning (18)	0.659 (0.323)	0.920		0.789
*D Effect*	3rd Thinning (17)	0.668 (0.325)	0.921		0.790
*E Effect*	4th Thinning (16)	0.771 (0.330)	0.928		0.807

#### Linear mixed-model estimates of seedling vigor

**Figure 2:**
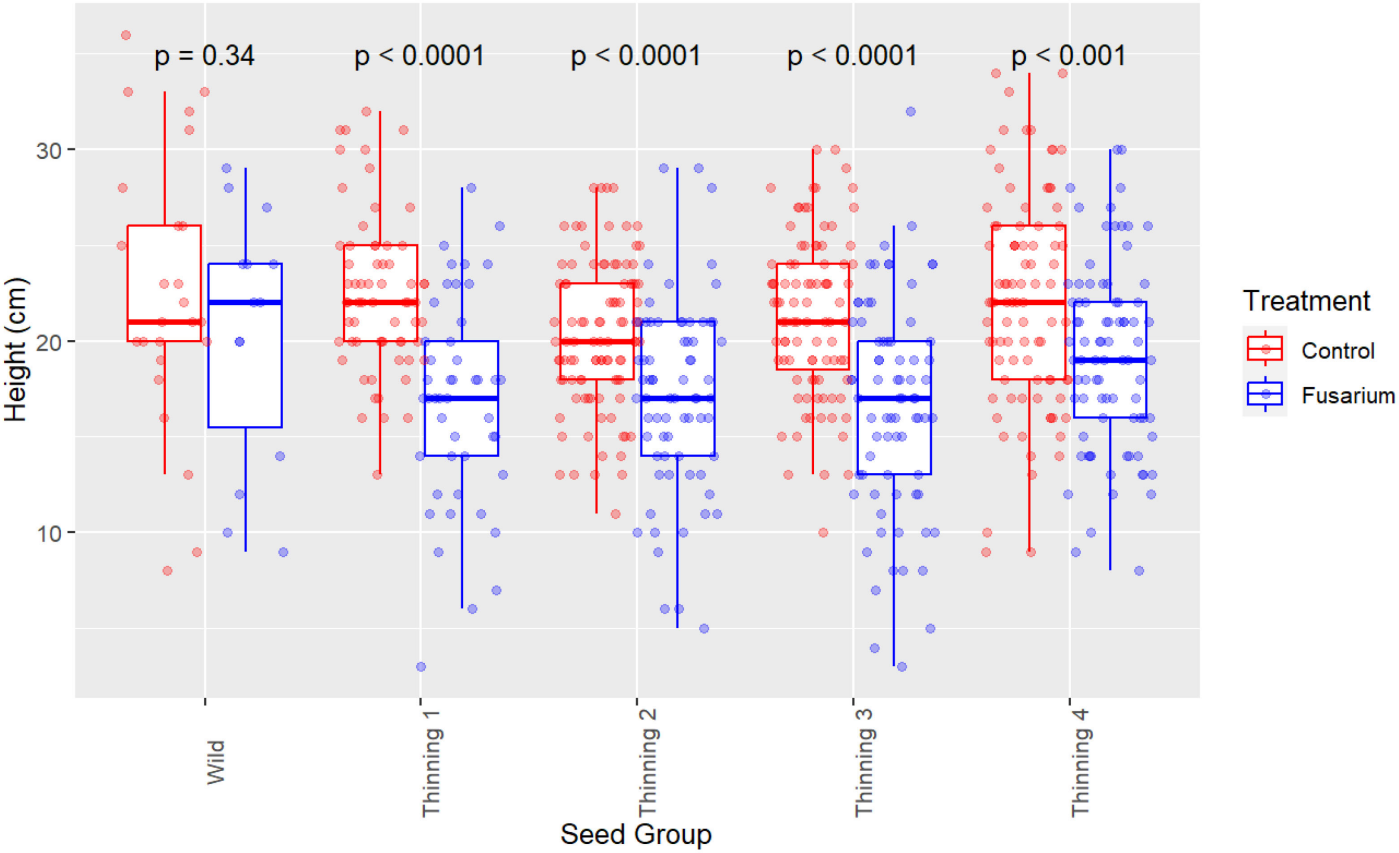
Seedling vigor (height) by seed source group. Comparison of control split (no FOXY applied) and fusarium (FOXY applied) tolerance. P-values were calculated using Welch’s Two-Sample t-test, comparing seedling height within each seed group between control and FOXY applied.

Survival proportion is not the only effect of FOXY on koa seedlings where vigor (seedling height) of remaining seedlings (end of trial) is diminished across all seed source groups (**Figure 2**). Welch’s two sample t-test identifies significant (p<0.0001) differences between the split – control versus FOXY applied, with mean height of control 21.53 (mm) and of FOXY treated 17.70 (mm). Linear mixed-model is used to estimate vigor (seedling height) at 120 days post germination in control (untreated) and fusarium (treated) conditions (**Table 1**). The unexpected trend identified in data exploratory visualization (**Figure 2**) is corroborated using mixed-model, where the surviving wild seed has the highest vigor estimate (19.28 mm in height) out of all groups with Group E (4^th^ thinning) as the next highest estimate (18.69 mm; **Table 1**). It can be inferred that this trend is exhibitive of disease tolerance in orchard groups where rather than fusarium causing death in seedlings (lack of resistance observed in wild seed; **Table 2**), seed derived from orchard groups have diminished growth and vigor because of the fitness cost to fight infection (**Table 1**).

### Stochastic simulation for projected gain under variable crossing parameters

**Figure 3:**
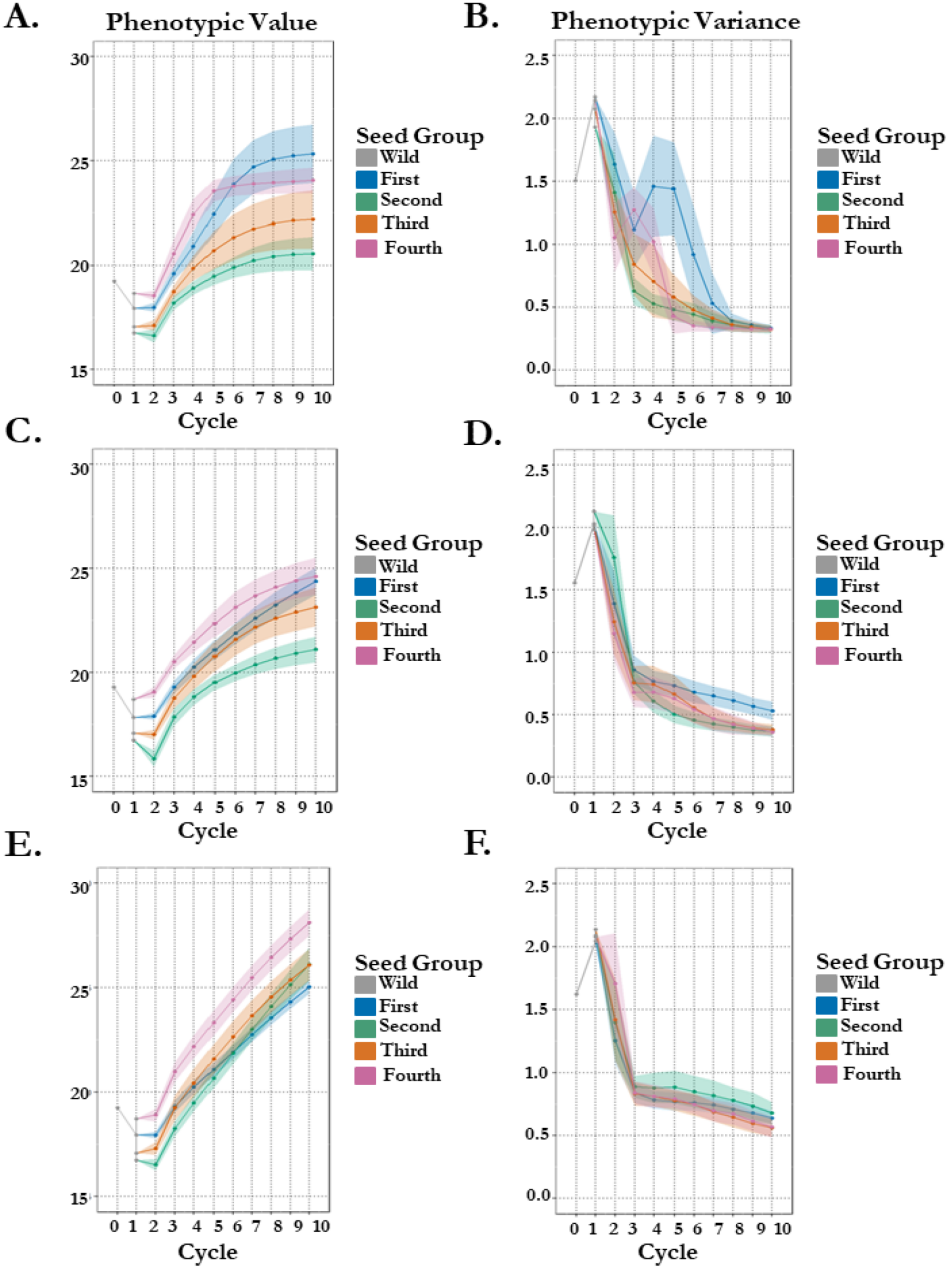
100 stochastic simulations of seedling vigor in koa with different crossing parameters and survivability by seed group origin. Phenotypes are estimated in 1 environment. (A/B) Genetic architecture of seedling vigor is simple oligogenic (8 QTL). Points represent the mean per cycle **(A)** of the 100 replications with standard error **(B)** representing standard deviation of replication outputs. (C/D) Genetic architecture of seedling vigor is complex oligogenic (20 QTL). Points represent the mean per cycle **(C)** of the 100 replications with standard error **(D)** representing standard deviation of replication outputs. (E/F) Genetic architecture of seedling vigor is polygenic (100 QTL). Points represent the mean per cycle **(E)** of the 100 replications with standard error **(F)** representing standard deviation of replication outputs.

Stochastic simulation was used to project gain in seedling vigor with variable seedling survival via FOXY resistance. We begin with parameters matching empirical estimates for each of our four orchard seed groups to project gain in vigor through 10 cycles of selection with varying genetic architecture complexity. Simple oligogenic control (8 QTL) of seedling vigor finds a rank change in the top two vigor estimate seed groups (4^th^ thinning: 18.69 & 1^st^ thinning: 17.91) after 10 cycles of selection (**Figure 3A**). The 1^st^ thinning seed group gains phenotypically 41% over 10 cycles of selection, compared to 29% in the 4^th^ thinning seed group. This is a likely result of more genetic variation within the 1^st^ thinning seed group (43 breeding parents). These groups have a projected split at generation 6, where standing variation in each population has diminished by 84% in the 4^th^ thinning group versus 58% in the 1^st^ thinning group (**Figure 3B**). Increase in genetic complexity to complex oligogenic (20 QTL) of the simulated trait finds no overlaps in genetic gain in 10 cycles of selection with seed groups ranked in accordance with estimates used (**Figure 3C**). It appears the 1^st^ thinning seed group will overtake the 4^th^ thinning seed group in the 11^th^ cycle, especially considering the loss of genetic variation is almost 10% greater in the 4^th^ thinning group, meaning more genetic variation for selection is present in the 1^st^ thinning seed group (**Figure 3D**). Another increase in genetic complexity to polygenic (100 QTL) of the simulated trait finds overlaps in genetic gain in 10 cycle of selection with seed groups ranked according to their survival probabilities, with the worst in gain being the first (67%) and the best in gain being the fourth seed group (81%), highlighting the potential for disease resistance to maintain genetic variation becomes more important to gain in primary traits as the architecture of those traits becomes increasingly complex (**Figures 3E, F**). The effect of a less complex trait, disease tolerance, on the gain in a complex trait, vigor (simulated: 100 QTL), raises some questions regarding whether this effect may be mitigated through breeding population size.

**Figure 4:**
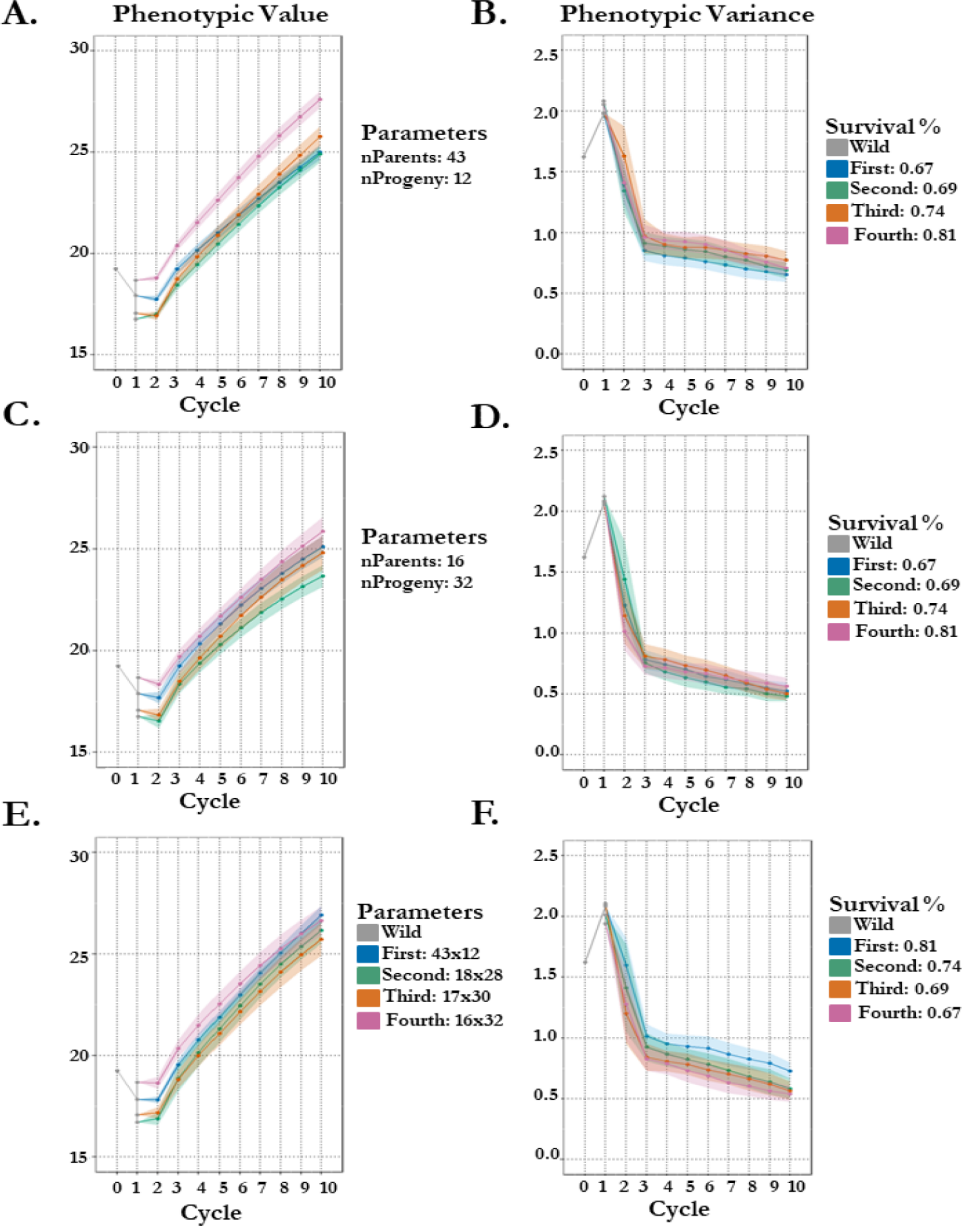
100 stochastic simulations of seedling vigor in koa with altered schemes by different crossing parameters and survivability by seed group origin. Phenotypes are estimated in 1 environment. Genetic architecture of seedling vigor is simulated polygenic (100 QTL). (A/B) Scheme 1: seed group survival % is same as empirical estimates while number of parents and progeny are set constant, 43 and 12 respectively. Points represent the mean per cycle **(A)** of the 100 replications with standard error **(B)** representing standard deviation of replication outputs. (C/D) Scheme 2: seed group survival % is same as empirical estimates while number of parents and progeny are set constant, 16 and 32 respectively. Points represent the mean per cycle **(C)** of the 100 replications with standard error **(D)** representing standard deviation of replication outputs. (E/F) Scheme 3: seed group survival % is inverted from empirical while number of parents and progeny is same as empirical. Points represent the mean per cycle **(E)** of the 100 replications with standard error **(F)** representing standard deviation of replication outputs.

We therefore implement 3 different schemes by altering either seed group crossing parameters (number of parents and progeny) and/or survival probability. The first scheme was designed to test the effect of survival probability on projected gain when using the different seed groups (differ by cycle 1 estimates: **Table 1**) while maintaining a larger breeding population size (43 parents). Here we observe increases in final gain comparative to the seed groups survival probability: First (67%) gains 7.06 mm in height, Second (69%) gains 8.14 mm, Third (74%) gains 8.73 mm, and Fourth (81%) gains 8.92 mm (**Figure 4A**). Moreover, this change in breeding population size from 16 parents to 43 parents increases variance in the 4^th^ thinning seed group by almost 10% in the 10^th^ cycle of selection (**Figure 4B**). The second scheme was designed to test the effect of survival probability on projected gain when using a smaller breeding population size (16 parents) for each seed group.

Results of gain are more variable in the second scheme, but seed groups finish 10 cycles of selection in the same order as their cycle 1 estimates (**Table 1**; **Figure 4C**). The more noticeable effect of the decrease in breeding population size is the loss of variance, where the 10^th^ cycle for each seed group has more variation in the second compared to in the first scheme: First loses 67% versus 75%, Second loses 66% versus 77%, Third loses 61% versus 76%, and Fourth loses 66% versus 73% (**Figures 4B, D**). The third scheme was designed to test whether the effect of survival has a proportional effect to the variable breeding population sizes used empirically. Despite differing starting points (seed group vigor estimates; **Table 1**), when survival probabilities are inverted (decreasing) and breeding population parameters are kept the same as empirical (decreasing), final projected gain of height in mm across all four groups is almost within 1 unit (First: 26.92 mm & Third: 25.73 mm; **Figure 4E**). More variation (30%) is present in the first seed group breeding population than the alternatives, highlighting the importance of a large breeding population size (43 parents) and high degree of disease resistance (81%) (**Figure 4F**).

## Discussion

### Trait associations and influences on gain of vigor

Seedling attributes such as height, root mass, abiotic and biotic resistances, and nutrient status are widely recognized to be critical components of plant success, especially in perennial tree species (Grossnickle and MacDonald, 2018). Improving genetic gain of vigor, measured as seedling height in our study, is especially important in forestry, where the cycle from planting to harvest is extended. Vigor can be selected at early stages and decrease the breeding cycle time as identified in *Acacia mearnsii* De Wildeman (Bisognin et al., 2023). Although Bisognin et al. (2023) identified substantial residual variance accounting for *A. mearnsii* height, the heritable fraction permits operational efficiencies in their program, similarly to the efficiencies sought by the *A. koa* program. Furthermore, selections in the *A. mearnsii* program exhibit an 85% increase to relative performance by early selection of tree vigor, thereby confirming the methods of selecting for tree vigor within the nursery. Orchard stand and yield potential is directly impacted by early-stage vigor as well as stress resistance. This has been observed in the contemporary crops cotton and alfalfa. For example, more vigorous cotton seedlings have a shortened duration of sensitivity to pathogens by the reduction of fungal penetration into developed woody tissue (Bourland, 2019). There also is a slight negative correlation between fusarium wilt in alfalfa and vigor, indicating that selection for fusarium wilt resistance might increase vigor even in the absence of disease (Fonseca et al., 1999). Additionally, vigor is an important indicator of yield potential, as observed in wild soybean (Kofsky et al., 2020). These patterns are also observed in forestry, where in southern pine production, seedling quality and vigor play a critical role in survivability and growth potential (Johnson and Cline, 1991; South et al., 2001), also observed in resistant eco-types of koa (Dudley et al., 2015, 2020). South et al. (2001) observed in southern (loblolly) pine an interaction between early growth and seedling size which results in greater establishment intensity, pointing to another trait which the *A. koa* breeding program can focus efforts on. Moreover, koa seedlings planted in Kokee with larger tree height and diameter come from families (PH4 & PH10) with an increased survival to FOXY (Dudley et al., 2020). The improvement of vigor, which is entangled with resistance, indicates the progress of population improvement during incipient domestication. The selection of FOXY resistant types and removal of susceptible genotypes from the breeding population results in observed improvements of both traits in koa (**Table 1**, **2**). Our breeding population continues to generate more vigorous and resistant progeny through this selection regime. Moreover, simulation predicts the population to maintain this progress of seedling vigor improvement (**Figure 4**). However, simulation does identify optimal crossing parameters given our population and genetic architecture, where maintaining a larger breeding population and having fewer progeny per cross (Scheme 1; **Figure 4A**) will improve gain substantially over alternative schemes (**Figures 4C, E**). Each generation consists of more vigorous and resistant progeny, pushing the population towards a domesticated state which will successfully fill the cultural, economic, and ecological role of wild koa.

### Bottleneck size, genetic load, and effects on LD

Genetic variation is an extremely important consideration during neo-domestication, where historical domestication incurred substantial bottlenecks (Eyre-Walker et al., 1998; Zhu et al., 2007; Allaby et al., 2008). Moreover, loss of variation during selection limits the potential for sustainable quantitative trait improvement (Lande, 1979). Our case study into koa domestication extends the important considerations to plasticity and adaptability. The improved types are not only for the agroecosystem but also for reforestation efforts, where a narrow-genetic base could spell abiotic and biotic disaster considering the worsening climatic conditions and expanding global trade (Foley et al., 2011; Ramankutty et al., 2018; Chapman et al., 2017). As we begin to bottleneck our population during domestication, consideration of realized and masked genetic load is important. Bottlenecks purge some deleterious mutations (reducing load) but it also converts masked load into realized load with prolonged bottlenecks fixing deleterious mutations (Bertorelle et al., 2022). The ratchet effect is a common cost of domestication as regions of low effective recombination, often the selected haplotypes, disproportionately accumulate deleterious variants (Kono et al., 2016). Balance can be restored using migration (genetic rescue), a component worth consideration in breeding scheme development for neo-domestication. Our breeding schemes (**Figure 4**) highlight the influence of bottleneck size on genetic variation after 10 cycles of selection, where Scheme 1 (43 parents) possesses ~25% more variation than Scheme 2 (16 parents). However, it should be expected that following incipient domestication effective population sizes become small, like crop species, creating a strong prevalence of genome-wide linkage disequilibrium that should validate the use of genomic selection (Voss-Fels et al., 2019). Careful consideration is necessary though because high LD decay is expected during the bottlenecking of an outbred species with high effective population size, reducing the predictive ability of genomic selection (Zhang et al., 2016).

### Limitations

This study focused on testing breeding program efficiencies through parsing the effects of different thinning stages/seed groups - seed harvested from decreasing orchard size and improved orchard traits - on vigor and resistance to inform seed production protocols for agricultural and conservation efforts. Considering this predetermined objective, half-sibling seeds were bulked by thinning stages, preventing further genetic analysis at the family level. Furthermore, stochastic simulation is useful for testing hypotheses and elucidating program efficiencies. Thus, the tested schemes represent a small fraction of all possible iterations and were chosen given different program constraints.

## Conclusion

In the koa example, large improvements are realized in a few cycles of selection even despite the long temporal time of this perennial hardwood tree species. Our initial selection on agronomic traits has empirically improved the adaptability of our populations to the agricultural and natural ecosystem. These improvements in adaptation to the agroecosystem highlight these agronomic traits as important domestication syndrome for koa. Furthermore, orchard stand is improved, meaning the breeding program can shift to quality traits such as rich and complex fiddleback grain. The stochastically simulated breeding schemes outline rapid domestication when traits of interest are known. Moreover, the speed of this change in phenotypic performance occurred regardless of the underlying genetic architecture, giving promise to the introduction of new crops with alternative domestication syndromes. Our empirical and simulated evidence of the trend towards domestication in *Acacia koa* outlines parametrization of crossing in the breeding cycle for rapid improvement of adaptation to the agroecosystem.

## Data Availability

The datasets presented in this study can be found in online repositories. The names of the repository/repositories and accession number(s) can be found below: https://github.com/Nfumia.

## Supplementary material

Supplementary Figure 1 Koa wild collection, pedigree, and operational diagram to illustrate the process of deriving thinning group seedling collections.

Supplementary Table 1 PCR primer sequences used in this study. All primers used the following conditions. Mastercycler ProS thermocycler and a PCR cycle program of 94°C for 2 min, 40 cycles of 94°C for 40 s, 58°C for 40 s, and 72°C for 30 s, and 72°C for 5 min. Products were run on a 1.5% agarose gel to visualize amplified PCR product using GelRed^®^.

Supplementary Table 2 Molecular identification of *Fusarium oxysporum f.* sp. *Koae*. Seed groups refer to the source of the seed (A: Wild Seed, B: Thinning 1, C: Thinning 2, D: Thinning 3, and E: Thinning 4), split refers to whether the seedling tested was control (sterile media) or treated (FOXY inoculum), the number tested is the number of individual plants sent for hyphal growth and sequencing, and FOXY identified is the percentage of the number tested that were positively identified as FOXY.
