# Supplementary material for: Acacia koa seedling disease tolerance and vigor driven by breeding orchard size
Source: Front Plant Sci. 2025 May 8;16:1544491. doi: 10.3389/fpls.2025.1544491 (PMC12095169; doi:10.3389/fpls.2025.1544491)
Supplement: Supplementary Figure 1 — Koa wild collection, pedigree, and operational diagram to illustrate the process of deriving thinning group seedling collections. [file DataSheet1.docx]

**Supplemental Figure 1.** Koa wild collection, pedigree, and operational diagram to illustrate the process of deriving thinning group seedling collections.


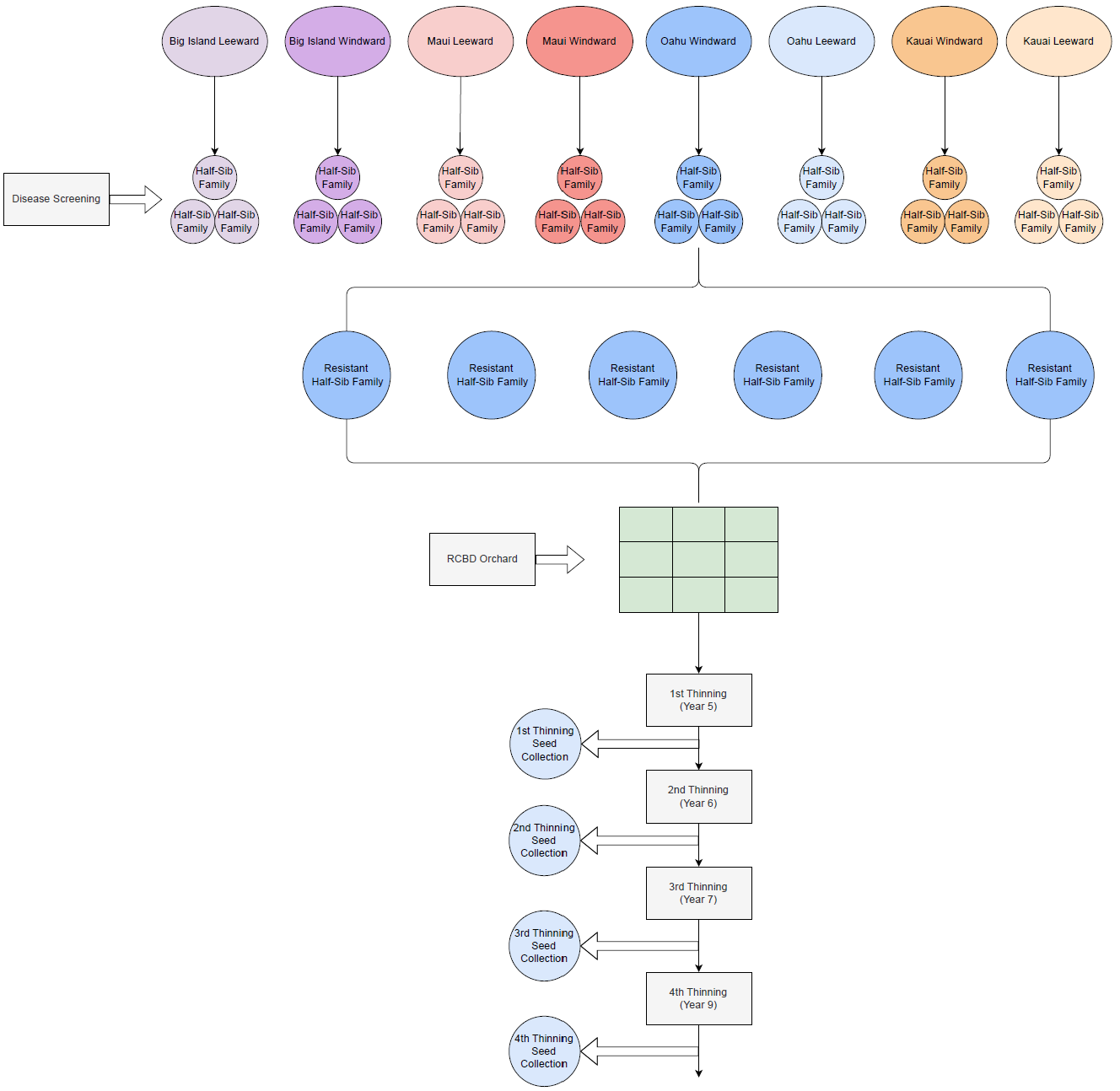


**Supplemental Table 1.** PCR primer sequences used in this study. All primers used the following conditions. Mastercycler ProS thermocycler and a PCR cycle program of 94 °C for 2 min, 40 cycles of 94 °C for 40 s, 58 °C for 40 s, and 72 °C for 30 s, and 72 °C for 5 min. Products were run on a 1.5% agarose gel to visualize amplified PCR product using GelRed®.


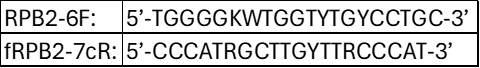


#

**Supplemental Table 2.** Molecular identification of *Fusarium oxysporum f. sp. Koae*. Seed groups refer to the source of the seed (A: Wild Seed, B: Thinning 1, C: Thinning 2, D: Thinning 3, and E: Thinning 4), split refers to whether the seedling tested was control (sterile media) or treated (FOXY inoculum), the number tested is the number of individual plants sent for hyphal growth and sequencing, and FOXY identified is the percentage of the number tested that were positively identified as FOXY.


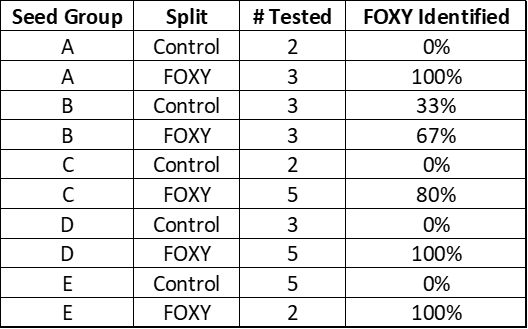


# 
